# Inference of transcription modification in long-live yeast strains from their expression profiles

**DOI:** 10.1186/1471-2164-8-219

**Published:** 2007-07-06

**Authors:** Chao Cheng, Paola Fabrizio, Huanying Ge, Valter D Longo, Lei M Li

**Affiliations:** 1 Molecular and Computational biology program, Department of Biological Sciences, University of Southern California, Los Angeles, CA 90089-2910, USA; 2 Andrus Gerontology Center and Department of Biological Sciences, University of Southern California,3715 McClintock Avenue, Los Angeles, CA 90089, USA; 3 Department of Mathematics, University of Southern California, Los Angeles, CA 90089, USA

## Abstract

**Background:**

Three kinases: Sch9, PKA and TOR, are suggested to be involved in both the replicative and chronological ageing in yeast. They function in pathways whose down-regulation leads to life span extension. Several stress response proteins, including two transcription factors Msn2 and Msn4, mediate the longevity extension phenotype associated with decreased activity of either Sch9, PKA, or TOR. However, the mechanisms of longevity, especially the underlying transcription program have not been fully understood.

**Results:**

We measured the gene expression profiles in wild type yeast and three long-lived mutants: *sch9*Δ, *ras2*Δ, and *tor1*Δ. To elucidate the transcription program that may account for the longevity extension, we identified the transcription factors that are systematically and significantly associated with the expression differentiation in these mutants with respect to wild type by integrating microarray expression data with motif and ChIP-chip data, respectively. Our analysis suggests that three stress response transcription factors, Msn2, Msn4 and Gis1, are activated in all the three mutants. We also identify some other transcription factors such as Fhl1 and Hsf1, which may also be involved in the transcriptional modification in the long-lived mutants.

**Conclusion:**

Combining microarray expression data with other data sources such as motif and ChIP-chip data provides biological insights into the transcription modification that leads to life span extension. In the chronologically long-lived mutant: *sch9*Δ, *ras2*Δ, and *tor1*Δ, several common stress response transcription factors are activated compared with the wild type according to our systematic transcription inference.

## Background

The yeast *S.cerevisae *has become one of the most valuable model organisms for ageing studies. In this uni-cellular eukaryote, two distinct paradigms are used to measure longevity. The first, replicative life span (RLS) is defined as the mean or maximum number of daughter cells generated by individual mother cells [1]. The second, chronological life span (CLS) is a measure of the mean or maximum survival time of populations of non-dividing yeast [2]. Yeast RLS has been proposed as a model for the ageing of dividing cells of higher eukaryotes, whereas CLS is believed to better model the ageing of post-mitotic cells [3-5]. RLS was the first paradigm to be used for ageing studies. Currently about 50 genes have been implicated in determining RLS. In comparison, fewer genes have been shown to regulate the chronological ageing. Recent studies have indicated three nutrient responsive yeast kinases: Sch9, PKA, and TOR, as major regulators of both types of ageing. Sch9 is a yeast kinase homologous to mammalian serine/threonine protein kinase Akt. Inactivation of Sch9 increases RLS by 30–40% [6] and extends CLS by nearly three folds [4]. Down-regulation of PKA activity obtained by introducing mutations in *RAS2 *and *CYR1 *(encoding proteins that regulate PKA activity) approximately doubles the CLS of yeast [4,5]. Recently, two high-throughput screenings were performed in yeast to identify genes that determine RLS and CLS, respectively. The first screening identified 10 gene deletions that increase RLS, and 6 of them (including the deletion of *TOR1*) correspond to genes encoding proteins in the TOR pathways [7]. The other screening identified several TOR-related gene deletions that increase CLS [8]. In yeast, as well as in higher eukaryotes, Sch9, PKA, and TOR coordinate signals from nutrients to regulate ribosome biogenesis, stress response, cell size, autophagy, and other cellular processes [9-12]. Of more importance, mutations that decrease the activity of the orthologs of these proteins in higher eukaryotes also extend life span, suggesting that the roles of these kinases in the regulation of life span are conserved along evolution [13-17].

Although the roles of Sch9, PKA, and TOR on life span extension are not fully understood, it is known that some stress response genes down-stream of these pathways are required for longevity. In the *ras2*Δ cells, the CLS extension is mediated by stress resistance transcription factor Msn2 and Msn4, which induce the expression of genes encoding for several heat shock proteins, catalase (Ctt1) and superoxide dismutase (Sod2). Transcription regulation of these genes by Msn2/Msn4 depends on the existence of a stress response element (STRE) in their promoter regions [5]. Sod2 is required for life span extension in *ras2*Δ and *sch9*Δ and over-expression of Sod2 extends longevity [18]. Moreover, longevity in the *sch9*Δ cells depends on the activity of Rim15 kinase [4]. The kinase Rim15 is known to integrate signals from TOR, PKA, and Sch9 [19], and activates Gis1, a transcription factor, which regulates genes containing a PDS (postdiauxic shift) element and is involved in the induction of theromotolerance and starvation resistance by a Msn2/Msn4-independent mechanism [20].

**Figure 1:**
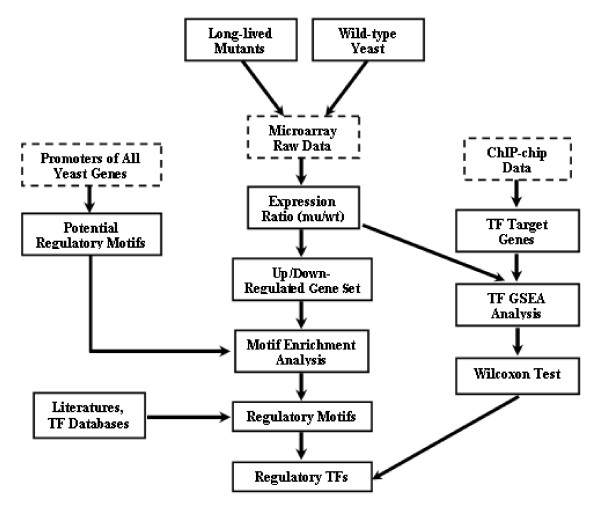
Scheme of transcriptional inference in the long-lived yeast mutants.

To better understand the function of Sch9, PKA and TOR kinases in yeast life span extension, we measured the gene expression profiles of wild type yeast as well as three long-lived mutants: *sch9*Δ, *ras2*Δ, and *tor1*Δ using the Affymetrix microarray technology. In this paper, we aim to address the question: what are the transcription factors that are involved in the longevity of these mutants? A number of methods have been proposed to answer this question. A straightforward method is to identity a set of differentially expressed or co-expressed genes, and then search their promoter sequences for known transcription factor binding sites or use de nova motif finding method to identify enriched motifs [21]. However, results obtained by this method are sensitive to the selection of the reference set, the cutoff value and some other factors. To overcome this problem, a systematic and statistical approach called PAP (promoter analysis pipeline) is proposed, which suggests an integrated model considering all of the promoters and characterized transcription factors in a genome [22]. Other two methods, REDUCER [23] and MOTIF REGRESSOR [24], identify regulatory motifs in response to a condition by associating log expression value of a gene with the motif abundance or motif-matching score in its promoter region using a linear model. In this paper, we apply two systematic strategies, as does PAP, to infer the regulatory transcription factor associated with longevity in *sch9*Δ, *ras2*Δ, and *tor1*Δ cells. The first strategy is based on motif analysis. We perform de novo identification of motifs from all the yeast promoter sequences and then test the enrichment of them in the up/down-regulated genes of long-lived mutants using gene expression in wild type yeast as control. The second strategy is based on the ChIP-chip data that measures the connectivity of transcription factors with genes. We seek the transcription factors that are significantly associated with up/down-regulated genes in the long-lived mutants. The schematic representation of our transcriptional inference in the long-lived yeast mutants is shown in Figure 1. According to our analysis, several transcription factors including Msn2/Msn4 and Gis1 are likely to function at the down-stream of the Sch9, PKA, and TOR pathways and may account for the longevity of the corresponding long-lived mutants. Furthermore, our analysis suggests that it is useful to combine microarray gene expression profiles with other data sources such as ChIP-chip data or promoter sequences to extract more biological information.

## Results and discussion

### Microarray data

We extract RNA samples from day 2.5 cells of wild-type as well as three long-lived yeast mutants: *sch9*Δ, *tor1*Δ, and *ras2*Δ, and measured expression levels of 5841 genes using the Affymetrix Yeast2.0 array. It should be noted that all these yeast strains are cultured in minimal medium SDC (synthetic dextrose complete)according to the standard methods for chronological life span measurement [2]. In the SDC medium, a substantial proportion of yeast cells are still dividing before day 2. At older ages, such as day 3–5, most of the cells become hypometabolic, which is associated with a dramatic drop in transcription. Therefore, we harvest mRNA at day 2.5 so that we can extract enough mRNA for microarray experiment while avoid the noise introduced by the transcriptional activities of dividing cells. We compute the log expression ratios for all the genes in each mutant with respect to the wild-type. The expression profile for *sch9*Δ, *tor1*Δ, and *ras2*Δ show strong similarity with one another, suggesting that Sch9, TOR, and PKA may control the expression of a common set of genes that are crucial for the chronological ageing.

### Motif enrichment analysis

We identify 539 putative regulatory motifs from sequences that include up to 800 bp upstream of all yeast genes. Among these putative motifs, 49 can be associated with a transcription factor according to literature or database [25]. Generally, if the activity of a transcription factor is changed as a consequence of some biological events, such as the deletion of *SCH9 *gene, we will expect to see an enrichment of its binding motif in the promoter regions of up-regulated or down-regulated genes. Motivated by this rationale, we performed enrichment analysis for these 539 putative regulatory motifs. First, we obtain the up-regulated and down-regulated gene sets for each mutant (with respect to the wild type) by setting the threshold to 1.8 fold. Then, for each of these gene set, we test the enrichment of a given motif in their promoter regions. These tests are carried out for all the 539 predicted motifs and a p-value of enrichment significance is assigned to each motif. Finally, we perform multiple testing corrections by calculating the corresponding q-values for all these enrichment tests.

In the up-regulated gene set for *sch9*Δ, *ras2*Δ, and *tor1*Δ (fold change with respect to wild type greater than 1.8), we identify 13, 5, and 8 enriched motifs out of the 537 motifs, respectively, at a significance level of 0.001 (q-value < 0.001). If we set the significance level to 0.01, the numbers of enriched motifs increase to 77, 43, and 43, respectively. Among these significant motifs, 14 can be associated with known transcription factors including Msn2/Msn4 and Gis1. In the down-regulated gene set for *sch9*Δ, *ras2*Δ, and *tor1*Δ (fold change with respect to wild type greater than -1.8), no motif is found to be enriched after multiple testing correction even at a significance level of 0.01. The predominance of enriched motifs in up-regulated gene sets suggests that the life span extension of these mutants is mediated by activation rather than repression of some transcription factors.

### Significantly enriched motifs

Table 1 shows the motifs that are enriched in the up-regulated gene set for at least one out of the three mutants and whose function or associated transcription factor is known. As shown, motifs associated with transcription factors Fhl1, Msn2/Msn4, and Gis1 are significantly enriched in up-regulated gene set for all the three long-lived mutants. The first transcription factor, Fhl1, is known to regulate the transcription of ribosomal protein (RP) genes via TOR and PKA in yeast [10]. In *sch9*Δ, *ras2*Δ, and *tor1*Δ, 233, 444, and 234 genes are up-regulated by at least 1.8 fold relative to wild type. Among them, 29, 27, and 27 are cytosolic RP genes, suggesting a significant enrichment of cytosolic RP genes in their up-regulated gene sets (p-values are 4.9E-15, 6.0E-7, and 3.4E-13, respectively). Although supported by the data, the up-regulation of RP genes is unexpected, considering that PKA and TOR are positive regulators of RP genes [10,12]. It is possible that RP gene expressions change along the chronological ageing process, and they are more expressed at day 2.5 in mutants compared the wild type. In addition, other factors than Fhl1 may be involved in the regulation of RP genes as well. The second transcription factor, Msn2 and the partially redundant factor Msn4, regulate the expression of many stress-responsive genes, including genes encoding heat shock proteins, catalase, and Sod2 [26,27]. Double deletion mutant *msn2msn4*Δ is highly sensitive to different stresses, including heat shock, carbon source starvation, and oxidative stresses. Activity of Msn2/Msn4 is negatively regulated by PKA kinase by nuclear exclusion [28-30]. Moreover, Msn2/Msn4 is required for the life span extension in mutations that decrease the activity of Ras2 (*ras2*Δ) or Cyr1 (cyr1::mTn) [4,5]. The third transcription factor, Gis1, is also negatively regulated by the PKA activity, and mediates gene expression during nutrient limitation [20]. Enrichment of motifs associated with Msn2/Msn4 and Gis1 in the up-regulated gene set suggests the important roles played by stress response genes in life span extension of the three long-lived mutants: *sch9*Δ, *ras2*Δ, and *tor1*Δ.

**Table 1:** Significantly enriched motifs with known transcription factors in the up-regulated gene sets

**Consensus Sequence**	**Transcription Factor**	**sch9Δ/wt**		**ras2Δ/wt**		**tor1Δ/wt**	
		**p-value**	**q-value**	**p-value**	**q-value**	**p-value**	**q-value**

RTGT-YGGRTG	FHL1	1.4E-07	2.1E-05	6.3E-05	0.0011	4.0E-08	9.4E-06
AGGGG	MSN2/MSN4	5.4E-08	1.1E-05	7.6E-05	0.0013	1.3E-07	2.1E-05
AWAGGGAT	GIS1	3.9E-05	9.3E-04	3.1E-04	0.0029	2.5E-05	7.2E-04
ARGGGG	MSN2/MSN4	0.0012	0.0065	0.0096	0.021	1.8E-04	0.0022
RYGWCASWAAW	SUM1	0.28	0.15	9.1E-04	0.0052	6.2E-04	0.0043
GACACAAAA	NDT80	0.0099	0.021	0.0082	0.019	0.0020	0.0084
GY-TSKCACGTG-G	PHO4	0.0039	0.012	2.0E-04	0.0023	0.0059	0.016
A-CACCC-TT	AFT1	0.0020	0.0082	0.0052	0.015	0.014	0.025
AMAA-TGTGG	MET4	0.24	0.14	4.6E-05	0.0010	0.029	0.038
CGCATMCCCCAC	MIG1	0.026	0.036	4.4E-04	0.0037	0.057	0.056
MWGTGTGGCR	MET31	0.046	0.050	7.7E-04	0.0048	0.059	0.058
RRTCACGTG	CBF1	0.59	0.25	2.9E-04	0.0028	0.076	0.067
GAW-TTCTAGAA	HSF1	0.029	0.038	0.0022	0.0090	0.23	0.13
ACCYT-AGGTT	ZAP1	0.13	0.096	1.4E-04	0.0018	0.24	0.14

Other than Fhl1, Msn2/Msn4 and Gis1 binding motifs, motifs associated with other transcription factors are also enriched in the up-regulated gene set of *sch9*Δ, *ras2*Δ or *tor1*Δ. For example, the binding motif of transcription factor Hsf1, which is also involved in stress response, is significantly enriched in *ras2*Δ (q-value is 0.0090) [31,32]; the binding motif of transcription factor Mig1, which is involved in glucose repression, is significantly enriched in *ras2*Δ (q-value is 0.0037) [33]; the binding motif of transcription factor Sum1, which is a dominant suppressor of mutant of silent information regulator genes, is significantly enriched in *ras2*Δ (q-values is 0.0052) and *tor1*Δ (q-values is 0.0043) [34,35]. Further studies of these transcription factors under the *sch9*Δ, *ras2*Δ, or *tor1*Δ background may shed new light on the mechanism of enhanced longevity in these mutants.

### Stability of enrichment analysis

To show the effect of threshold setting, we performed motif enrichment analysis using different threshold values for up-regulation and down-regulation. For a wide range of thresholds from 2-fold to 1.4-fold, our analysis achieves similar results. First, the total number of significantly enriched motifs in the up-regulated gene set does not change much with different thresholds. Secondly, we identify almost the same set of significant enriched motifs using different thresholds. Thirdly, we do not identify any significantly enriched motifs in the down-regulated gene sets using all these threshold values.

**Figure 2:**
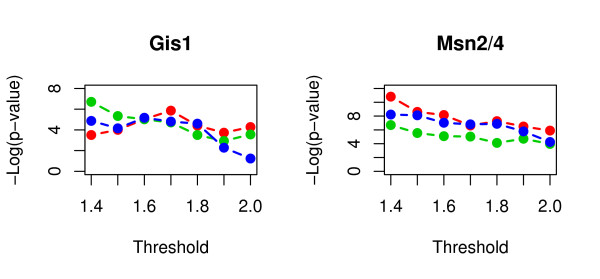
**Effect of cutoff value on enrichment analysis result**. The left and the right panel show the enrichment of Msn2/Msn4 and Gis1 in the up-regulated gene set in three long-lived mutants. The x-axis is the threshold for up-regulation. The y-axis is the negative log transformed p-value. The red, green, and blue line correspond to *sch9*Δ, *ras2*Δ, and *tor1*Δ mutant, respectively.

As shown in Figure 2, Gis1 and Msn2/Msn4 binding motifs exhibit significant enrichment in the up-regulated gene sets corresponding to different thresholds. Generally, a smaller threshold results in larger up- and down-regulated gene sets, based on which the enrichment analysis is more reliable and sensitive. As shown in Figure 2, the significance level of motif enrichment increases with the decrease of the threshold, suggesting a higher sensitivity for larger gene set. On the other hand, the threshold should be high enough to ensure that most genes in the up- or down-regulated gene set reflect real biological expression difference rather than background noises.

### ChIP-chip based analysis

In the motif enrichment analysis, those 539 motifs are identified from DNA sequence using a de novo method. The presence of a motif in the up-stream region of a gene is determined computationally and many of them may not be functional binding sites of transcription factors. For example, based on the computation, we find that 1849 out of the 5841 yeast genes have at least one Gis1 binding sites in their promoter (800 bp up-stream of translation initiation site) region. It is possible that the *in silico *method overestimates the number of genes regulated by a specific TF. If we know the target genes regulated by a transcription factor, we do not need to rely on computation methods for target gene identification and we may improve the accuracy of transcription inference.

The ChIP-chip experiment provides us with the information about the interaction between transcription factors and genes on a genomic scale. In yeast, the genomic occupancy of 203 transcription factors in rich media conditions was determined in a systematic ChIP-chip experiment [36]. For some of the transcription factors, their target genes in different experimental conditions, such as in heat shock, rapamycin treatment etc, were also determined. Based on the ChIP-chip data, we define 350 gene sets, each corresponding to a transcription factor under a specific condition. Among them, 203 gene sets correspond to target genes of these 203 transcription factors in YPD medium; the rest of them correspond to ChIP-chip results under other conditions. We performed gene set enrichment analysis (GSEA) for these 350 transcription factor gene sets (TF gene sets) [37]. GSEA analysis is a permutation based method to test if a set of genes tend to have high rank or low rank in a rank list, e.g. log expression rank list. In this work, we use GSEA analysis to test whether genes in a TF gene set tend to have high or low log expression values. By using this method, we identify 46 TF gene sets that are significantly enriched in at least one of the three long-lived yeast mutants at a FDR of 0.01. The accuracy of p-value assigned by GSEA analysis depends on the number of permutations which can not be too large considering the computation complexity. To obtain a more accurate p-value and to infer whether a gene set is positively or negatively affected, we carry out the Wilcoxon rank test to the 46 TF gene sets.

We compare the log expression values of genes in a TF gene set with the whole genome expression background (all the other genes) using the Wilcoxon rank test. In comparison with the wild type, if the target genes of a transcription factor tend to be up-regulated in a long-lived mutant according to the test, then we may infer that the activity of this transcription factor is positively affected in the mutant. Conversely, if the target genes of a transcription factor tend to be down-regulated, then we infer that the activity of this transcription factor is negatively affected. In total, we identify 29 positively affected TF gene sets involving 22 transcription factors from 7 ChIP-chip experiment conditions (see Table 2), and 6 negatively affected gene sets involving 5 transcription factors from 2 ChIP-chip experiment conditions (see Table 3). The ChIP-chip experiment conditions includes YPD (rich nutrient medium), H2O2Hi (highly hyperoxic, 4 mM H2O2), H2O2Lo (moderately hyperoxic, 0.4 mM H2O2), SM (amino acid starvation, 0.2 mg/ml sulfometuron methyl), Acid (acidic medium, 0.05 M succinic acid), RAPA (nutrient deprivation, 100 nM rapamycin), BUT14 (filamentation inducing, 1% butanol).

**Table 2:** Positively affected transcription factors in the long-lived mutants. The "num" column indicates the number of target genes of a transcription factor under a specific condition at the 0.01 significance level according to the ChIP-chip data.

**Transcription Factor**	**Condition**	**Num**	**sch9Δ/wt**		**rasΔ/wt**		**tor1Δ/wt**	
			**p-value**	**q-value**	**p-value**	**q-value**	**p-value**	**q-value**
CIN5	H2O2Hi	177	0.0022	0.012	7.5E-06	1.1E-04	0.0025	0.013
CIN5	H2O2Lo	315	4.5E-05	4.7E-04	2.4E-07	5.1E-06	0.0021	0.011
CIN5	YPD	274	0.0036	0.037	8.6E-07	3.5E-05	5.1E-04	0.0071
FHL1	H2O2Hi	188	0	0	8.8E-09	2.3E-07	2.2E-16	1.2E-14
FHL1	RAPA	214	0	0	5.6E-16	2.8E-14	0	0
FHL1	SM	287	0	0	1.5E-12	5.5E-11	0	0
FHL1	YPD	207	0	0	1.8E-14	1.7E-12	0	0
FKH2	H2O2Hi	331	0.0015	0.0087	1.3E-05	1.6E-04	1.4E-05	1.7E-04
GAT3	YPD	176	1.8E-14	1.7E-12	2.7E-08	1.4E-06	0	0
MET31	YPD	69	0.041	0.24	1.1E-04	0.0021	1.8E-04	0.0030
MET32	SM	93	1.4E-04	0.0012	1.8E-06	2.8E-05	1.4E-05	1.7E-04
MSN2	Acid	145	9.1E-04	0.0058	1.2E-05	1.6E-04	6.8E-07	1.2E-05
NDD1	YPD	190	0.037	0.23	2.8E-04	0.0041	8.2E-05	0.0017
NRG1	H2O2Hi	275	0.0087	0.035	5.5E-05	5.5E-04	0.088	0.22
NRG1	H2O2Lo	122	0.12	0.27	3.9E-06	5.8E-05	0.0081	0.033
PDR1	YPD	163	3.6E-06	1.3E-04	2.0E-04	0.0031	4.0E-06	1.3E-04
PUT3	H2O2Lo	156	4.7E-05	4.9E-04	2.4E-04	0.0019	0.0015	0.0086
RAP1	SM	387	0	0	5.7E-09	1.6E-07	1.1E-16	6.8E-15
RAP1	YPD	408	0	0	2.4E-10	1.6E-08	2.2E-16	3.2E-14
RGM1	YPD	107	2.3E-06	8.4E-05	3.1E-04	0.0045	4.0E-07	1.8E-05
RIM101	H2O2Lo	142	0.15	0.32	4.4E-04	0.0032	0.032	0.098
SFP1	SM	114	2.3E-15	9.9E-14	2.3E-07	5.1E-06	7.8E-16	3.6E-14
SMP1	YPD	181	1.2E-04	0.0022	0.0032	0.033	1.9E-04	0.0030
SOK2	BUT14	211	0.043	0.12	8.4E-05	7.9E-04	0.0045	0.020
STB1	YPD	89	0.16	0.61	0.036	0.23	3.6E-05	9.3E-04
SWI4	YPD	252	0.0074	0.070	8.9E-05	0.0018	1.4E-06	5.5E-05
XBP1	H2O2Lo	173	0.027	0.087	3.5E-05	3.8E-04	0.023	0.076
YAP5	YPD	167	1.7E-10	1.2E-08	3.7E-06	1.3E-04	6.7E-12	5.8E-10
YAP6	YPD	167	0.0037	0.038	1.6E-09	9.9E-08	1.0E-05	3.0E-04

**Table 3:** Negatively affected transcription factors in the long-lived mutants. The "num" column indicates the number of target genes of a transcription factor under a specific condition at the 0.01 significance level according to the ChIP-chip data.

**Transcription Factor**	**Condition**	**Num**	**sch9Δ/wt**		**ras2Δ/wt**		**tor1Δ/wt**	
			**p-value**	**q-value**	**p-value**	**q-value**	**p-value**	**q-value**
ABF1	YPD	549	1.9E-04	0.012	6.1E-07	9.6E-05	1.6E-07	3.2E-05
GCN4	RAPA	324	0.77	0.90	0.031	0.26	1.3E-09	4.2E-07
GCN4	YPD	143	0.16	0.43	0.0031	0.052	9.2E-11	3.6E-08
HAP4	YPD	126	4.2E-06	5.5E-04	0.0013	0.040	1.2E-05	0.0011
INO2	YPD	114	1.2E-05	0.0011	0.0038	0.056	0.0014	0.040
RTG3	RAPA	158	0.0016	0.038	9.9E-05	0.0059	1.3E-05	0.0010

Among the positively affected transcription factors shown in Table 2, many are related to stress or drug resistance, such as Msn2, Cin5, Pdr1, Smp1, Rim101, Yap6, and Xbp1. For example, Smp1, positively affected in all of these long-lived mutants especially in *sch9*Δ and *tor1*Δ, was reported to be involved in regulation of the response to osmotic stress [38,39]. Some cell cycle related transcription factors such as Fkh2, Stb1, Swi4 and Yap5 are also positively affected. Sok2, a negative regulator of cyclic AMP-dependent protein kinase [40,41], and Nrg1, a transcriptional repressor for glucose-repressed genes [42], are highly affected in *ras2*Δ and moderately affected in *sch9*Δ and *tor1*Δ, suggesting that they may act down-stream of Ras2, Sch9, and Tor1 to mediate the transcription response in low-nutrient environment. Consistently with the results from motif enrichment analysis, we again find Fhl1 to be positively affected in all the three mutants. Moreover, two additional transcription activators for ribosome genes, Rap1 and Sfp1, are also identified.

Despite that many transcription factors are positively affected in the long-lived mutants, we identify only a few negatively affected TF gene set using ChIP-chip based analysis (see Table 3), similar to the results by motif enrichment analysis. Hap4 forms a glucose-repressed complex with Hap2, Hap3, and Hap5, which functions as a global positive regulator of respiratory gene expression [43]. Rtg3 involves in the retrograde regulation in response to a mitochondrial defect [44]. Abf1 is a multi-functional global regulator for genes involved in a diverse range of cellular processes including carbon source regulation, nitrogen utilization, sporulation, meiosis, and ribosomal function [45,46]. Gcn4 activates the expression of amino acid biosynthetic genes in response to amino acid starvation [47]. The decrease of activity of these transcription factors might reflect the reduced respiration and low metabolic rate in the long-lived mutants [43-47]. In support of this hypothesis, we find many genes involved the oxidative phosphorylation and the TCA pathway (tricarboxylic acid cycle) are down-regulated. Interestingly, Rtg3 and Gcn4 are shown to be activated by TOR inhibition in previous studies [48,49]. We note that our inference is about association but not about causality by nature. The inconsistency of our results with previous studies may indicates that confounding factors exist and further investigation is necessary to understand the complete story.

### Comparison of the two methods

The results from motif based analysis and ChIP-chip based methods are roughly consistent with each other in the following way: (1) the transcription factors identified by motif based analysis tend to have small p-values in results of ChIP-chip based analysis, and vice versa; (2) both methods identify much more positively affected transcription factors, whereas no or only a few negatively affected transcription factors are identified; (3) both methods suggest stress response transcription factors may play important roles in the chronological life span extension of the mutants; (4) the transcription activator Fhl1 is found to be strongly associated with life span extension by both methods. On the other hand, there is also some inconsistency between the two methods, which may arise from the following reasons: (1) only about 50 motifs can be associated with known transcription factors according to literatures and transcription factor databases; (2) the binding information is not available for some transcription factors in the ChIP-chip data, such as Gis1; (3) The binding targets for some transcription factors are condition-dependent and none of the condition in the ChIP-chip data match our microarray experiment condition perfectly. As more data sets related to transcription factors are available, we would expect to improve substantially the accuracy of the analysis. For example, a perfect match between the conditions for microarray and ChIP-chip experiment would improve the results. We could infer the activity of more transcription factors by using the motif based method with the accumulation of binding information in the transcription databases, such as the TRANSFAC [50]. Fortunately, these kinds of data and information are accumulating rapidly and we can make more reliable inference based on new available data.

### Condition dependent of transcription factor binding

According to the genome-wide binding behavior across different conditions, transcription factors could categorized into four major classes: conditional invariant, conditional enabled, conditional expanded, and conditional altered transcription factors [36]. For conditional invariant transcriptional factors, their target gene are highly overlapped in different conditions and therefore the ChIP-chip result may be used to infer transcription for microarray data under other conditions. For example, Fhl1 is shown, according to our analysis, to be associated with expression changes in the *sch9*Δ, *ras2*Δ, and *tor1*Δ mutants, although the condition and cell status of the microarray and ChIP-chip experiments are quite different. However, for some other transcription factors, the transcription inference of their activity depends strongly on the experiment conditions. Table 4 shows the Wilcoxon test results for Msn2 and Msn4 target gene sets corresponding to different ChIP-chip experiment conditions. As can be seen, the number of binding targets of Msn2 and Msn4 changes dramatically in different conditions and consequently their activity changes in the long-lived mutants inferred by the Wilcoxon rank test are quite different. For example, Msn2 is inferred to be positively affected in *ras2*Δ based on the ChIP-chip result under Acid, H2O2Hi, and H2O2Lo conditions. But it is negatively affected, if the inference is based on ChIP-chip result under RAPA condition. For these reasons, results based on ChIP-chip data should be carefully examined, but in at least two situations it would provide valuable information for transcription inference: (1) when the transcription factor of interest binds with a relatively invariant set of target genes in different conditions; (2) when the experiment conditions for ChIP-chip and microarray experiment are equivalent or similar.

**Table 4:** Transcription inference of Msn2 and Msn4 based on ChIP-chip binding data from different conditions. Positive and negative effect is shown in normal and italic font, respectively. The "num" column indicates the number of target genes of a transcription factor under a specific condition at the 0.01 significance level according to the ChIP-chip data.

**Transcription Factor**	**Condition**	**Num**	**sch9Δ/wt**	**ras2Δ/wt**	**tor1Δ/wt**
MSN2	YPD	43	*0.33*	*0.04*	0.43
	Acid	145	9.1E-04	1.2E-05	6.8E-07
	RAPA	101	*0.14*	*0.033*	*0.24*
	H2O2Hi	114	0.014	0.0031	0.016
	H2O2Lo	140	0.18	0.004	0.019
	HEAT	24	*0.01*	*0.14*	*0.37*
MSN4	YPD	145	8.5E-04	0.21	7.9E-06
	Acid	37	0.13	0.018	0.067
	RAPA	131	*0.19*	*0.14*	*0.31*
	H2O2Hi	169	0.084	6.7E-04	0.033
	H2O2Lo	67	0.19	0.0036	0.048

## Conclusion

We have demonstrated how to infer the activity modification of transcription factors in the long-lived mutants with respect to wild-type yeast by integrating microarray expression data with promoter sequence data and ChIP-chip data. Both the motif and ChIP-chip data based analysis suggest that some transcription factors related to stress response or ribosomal genes may play important roles in the yeast chronological ageing. Interestingly, based on our analysis, the activities of Msn2/Msn4 and Gis1 are positively regulated in all the three mutants: *sch9*Δ, *ras2*Δ, and *tor1*Δ, which is consistent with previous studies. Moreover, we find some other interesting transcription factors that may also involve in the transcription regulation at the downstream of Sch9, PKA and TOR pathways. Finally, our analysis provides a framework for transcription inference by integrating microarray data with other data sources.

## Methods

### Microarray experiment and data processing

Gene expression in four yeast strains, wild type, *sch9*Δ, *ras2*Δ, and *tor1*Δ, is measured using DNA microarray analysis. Yeast cells from all strains are cultured in nutrient limited SDC (synthetic dextrose complete) medium, collected at day 2.5, then used to extract total RNA by the acid phenol method. Total RNA from independent cultures of each strain is used as a template to synthesize complementary RNA (cRNA). The cRNA is hybridized to Affymetrix GeneChip Yeast 2.0 Array to obtain the measurement of gene expression. For each strain, the experiment is repeated for 3 times, each obtained from independent population of corresponding strain. The Bioconductor Affy Package is adopted to process the microarray data [51]. The "Invariant Set" approach is used for normalization at the probe level, and the "Model based" method is used to summarize and obtain expression for each probe set [52]. High consistency is achieved between the replicates from the same strain, with the Pearson correlation coefficients greater than 0.96 at the gene level.

The Yeast 2.0 Array contains probe sets for both two yeast species: *S.cerevisiae *and *S.pombe*. Probe sets for *S.pombe *are excluded and only probe sets for *S.cerevisiae *are considered in later analysis. To calculate the gene expression change between two strains (each has 3 replicates), we compute the fold change for each pair of comparison. 3 × 3 comparisons result in 9 ratios, which are average to get the mean fold change (FC) of each probe set. For all the *S.cerevisiae *probe sets, the mean FC is calculated in three comparisons: *sch9*Δ/wt, *ras2*Δ/wt, and *tor1*Δ/wt. Most genes are represented each by a single probe set in Yeast 2.0 Array. For genes represented by more than one probe sets, we average the mean FCs of probe sets associated with them to obtain the gene level expression change.

### Motif identification and prediction

To obtain the potential regulatory motifs, we refer to the methods used by Beer et al. and downloaded the motif data from [53]. Beer et al. identified the significantly enriched motifs in the promoter regions of all yeast genes using AlignACE software [54,55]. The promoter region was defined as the DNA sequence from translation initiation site up to 800 bp upstream. Based on their motif data, we identify 539 significant motifs from the promoter regions after removing the redundancy. Among these motifs, 48 are associated with known transcriptional factors according to literatures.

### Motif enrichment analysis

We use the so-called motif enrichment analysis to identify the motifs associated with gene expression change between two yeast strains, i.e. *sch9*Δ/wt. If a motif is indeed related to expression change in *sch9*Δ/wt, for example, as a consequence of activation/repression of its associated transcription factor in *sch9*Δ, we would expect to see the enrichment of genes with the motif in the up/down- regulated gene set. Specifically, we use the Fisher exact test to identify significant enriched motifs in a up/down- regulated gene sets.

Suppose among all the *N *yeast genes, *M *genes contain a pre-defined motif, the remaining *N *- *M *genes do not contain this motif. We denote *X *as the number of genes that contain a given motif in a gene set of size *K*, and *X *follows a Hypergeometric distribution. That is,

p(M,N−M,x)=(Mx)(N−MK−x)(NM).
 MathType@MTEF@5@5@+=feaafiart1ev1aaatCvAUfKttLearuWrP9MDH5MBPbIqV92AaeXatLxBI9gBaebbnrfifHhDYfgasaacH8akY=wiFfYdH8Gipec8Eeeu0xXdbba9frFj0=OqFfea0dXdd9vqai=hGuQ8kuc9pgc9s8qqaq=dirpe0xb9q8qiLsFr0=vr0=vr0dc8meaabaqaciaacaGaaeqabaqabeGadaaakeaacqWGWbaCcqGGOaakcqWGnbqtcqGGSaalcqWGobGtcqGHsislcqWGnbqtcqGGSaalcqWG4baEcqGGPaqkcqGH9aqpdaWcaaqaamaabmaabaqbaeqabiqaaaqaaiabd2eanbqaaiabdIha4baaaiaawIcacaGLPaaadaqadaqaauaabeqaceaaaeaacqWGobGtcqGHsislcqWGnbqtaeaacqWGlbWscqGHsislcqWG4baEaaaacaGLOaGaayzkaaaabaWaaeWaaeaafaqabeGabaaabaGaemOta4eabaGaemyta0eaaaGaayjkaiaawMcaaaaacqGGUaGlaaa@49B2@

For each motif we calculate the p-value defined as *Pr*(*X *≥ *x*|*M*, *N *- *M*), which is the probability of observing x or more genes with the motif in their promoter regions. We test the significance of enrichment for all the 539 motifs in the up- and down-regulated gene sets of *sch9*Δ/wt, *ras2*Δ/wt, and *tor1*Δ/wt. To correct for the multiple testing, we compute the q-value using the "qvalue" package for R software [56].

### GSEA analysis

Gene set enrichment analysis is an approach to testing whether a set of genes, as a whole, are up-regulated or down-regulated compared to other gene sets [37]. Give an expression profile, we rank the log expression value of all the genes e_*i*_, *i *= 1, ... *N *in the decreasing order as denoted by L = {*g*_1_, *g*_2_, ... , *g*_*N*_}, where *N *is the total number of genes in the list. Then we evaluate the fraction of genes in a gene set *S *of size *N*_*H *_(hit) weighted by absolute expression values, and the fraction of genes not in S (missing) present up to a given position i in the list *L *as following:

$$\begin{array}{c} \begin{array}{cc} P_{hit}=\sum_{g_{j}\notin S,j\leq i}\frac{\left|e_{j}\right|}{N_{R}}, & where\;N_{R}=\sum_{g_{j}\notin S}\left|e_{j}\right|, \end{array}\\ P_{miss}=\sum_{g_{j}\notin S,j\leq i}\frac{1}{N-N_{H}}. \end{array}$$

The maximum deviation from zero of *P*_*hit *_- *P*_*miss *_is defined as enrichment score (ES) for the gene set S. Finally, the expression profile is permutated for many times to compute permutated ES values and the significance is determined by the percentage of permutated ES values that are larger or equal to the real ES value. In this paper, we do 10,000 permutations to estimate the significance of enrichment for a gene set. Again q-values are calculated for multiple testing correction.

### ChIP-chip based analysis

The ChIP-chip data are available from [57]. They contain DNA binding information for 203 yeast transcription factors, where each TF-gene association is assigned a P-value. It is noted that the binding information are only available for these 203 TFs under the YPD condition. For some of the transcription factors, binding information under other conditions are also available. Based on the ChIP-chip data, we define 350 TF gene sets: *S*_1_, *S*_2_, ..., *S*_350_, each containing the target genes of a transcription factor under a condition at the 0.01 significance level. To infer whether a transcription factor is significantly affected, we first perform GSEA analysis for these 350 TF gene sets using the above described methods. Then for the significant gene sets resulting from GSEA analysis, we further compare the gene expression of its associated gene set *S*_*i *_with the background gene expression denoted by *G *- *S*_*i*_, where *G *is the gene set containing all the yeast genes. We use the Wilcoxon rank test to calculate the p-value (one sided). If the expression levels of a gene set are significant higher than the background expression, we denote the corresponding transcription factor as positively affected. Similarly, if the expression level of a gene set is significant lower than the background expression, we denote the corresponding transcription factor as negatively affected.

## Authors' contributions

CC wrote the code, carried out the analysis, and drafted the manuscript. PF carried out the microarray experiment. HG was involved in data analysis. VL participated in design and coordination of the study. LL participated in design, coordination and writing of the study. All authors read and approved the final manuscript.
